# Investigation of the Heteroepitaxial Process Optimization of Ge Layers on Si (001) by RPCVD

**DOI:** 10.3390/nano11040928

**Published:** 2021-04-06

**Authors:** Yong Du, Zhenzhen Kong, Muhammet S. Toprak, Guilei Wang, Yuanhao Miao, Buqing Xu, Jiahan Yu, Ben Li, Hongxiao Lin, Jianghao Han, Yan Dong, Wenwu Wang, Henry H. Radamson

**Affiliations:** 1 Key Laboratory of Microelectronic Devices & Integrated Technology, Institute of Microelectronics, Chinese Academy of Sciences, Beijing 100029, China; duyong@ime.ac.cn (Y.D.); kongzhenzhen@ime.ac.cn (Z.K.); miaoyuanhao@ime.ac.cn (Y.M.); xubuqing@ime.ac.cn (B.X.); yujiahan@ime.ac.cn (J.Y.); linhongxiao@ime.ac.cn (H.L.); hanjianghao@ime.ac.cn (J.H.); dongyan2019@ime.ac.cn (Y.D.); wangwenwu@ime.ac.cn (W.W.); 2 Institute of Microelectronics, University of Chinese Academy of Sciences, Beijing 100049, China; 3 Department of Materials and Nano Physics, School of Information and Communication Technology, KTH Royal Institute of Technology, Isafjordsgatan 22, Kista, SE-164 40, Sweden; toprak@kth.se; 4 Research and Development Center of Optoelectronic Hybrid IC, Guangdong Greater Bay Area Institute of Integrated Circuit and System, Guangzhou 510535, China; liben@giics.com.cn; 5 Department of Electronics Design, Mid Sweden University, Holmgatan 10, 85170 Sundsvall, Sweden

**Keywords:** Ge, optimization, parameter, threading dislocation, strain, RPCVD

## Abstract

This work presents the growth of high-quality Ge epilayers on Si (001) substrates using a reduced pressure chemical vapor deposition (RPCVD) chamber. Based on the initial nucleation, a low temperature high temperature (LT-HT) two-step approach, we systematically investigate the nucleation time and surface topography, influence of a LT-Ge buffer layer thickness, a HT-Ge growth temperature, layer thickness, and high temperature thermal treatment on the morphological and crystalline quality of the Ge epilayers. It is also a unique study in the initial growth of Ge epitaxy; the start point of the experiments includes Stranski–Krastanov mode in which the Ge wet layer is initially formed and later the growth is developed to form nuclides. Afterwards, a two-dimensional Ge layer is formed from the coalescing of the nuclides. The evolution of the strain from the beginning stage of the growth up to the full Ge layer has been investigated. Material characterization results show that Ge epilayer with 400 nm LT-Ge buffer layer features at least the root mean square (RMS) value and it’s threading dislocation density (TDD) decreases by a factor of 2. In view of the 400 nm LT-Ge buffer layer, the 1000 nm Ge epilayer with HT-Ge growth temperature of 650 °C showed the best material quality, which is conducive to the merging of the crystals into a connected structure eventually forming a continuous and two-dimensional film. After increasing the thickness of Ge layer from 900 nm to 2000 nm, Ge surface roughness decreased first and then increased slowly (the RMS value for 1400 nm Ge layer was 0.81 nm). Finally, a high-temperature annealing process was carried out and high-quality Ge layer was obtained (TDD=2.78 × 10^7^ cm^−2^). In addition, room temperature strong photoluminescence (PL) peak intensity and narrow full width at half maximum (11 meV) spectra further confirm the high crystalline quality of the Ge layer manufactured by this optimized process. This work highlights the inducing, increasing, and relaxing of the strain in the Ge buffer and the signature of the defect formation.

## 1. Introduction

With the increasing use of optical fibers in telecommunication bands, there is a pressing need for low cost and high efficiency photodetectors working at the wavelength ranging from 1.3–1.55 μm. High-quality Ge film on Si has great potential to be used in the above-mentioned photodetectors owing to its special band structure [1,2,3,4,5]. Moreover, high-quality Ge film on Si can also be used as the feasible high–mobility channel material for high electron mobility devices [6,7,8,9], such as metal oxide semiconductor field-effect transistor (MOSFETs), fin field effect transistor (FinFET), nanowire devices, etc. [10,11,12,13]. Meanwhile, Ge has been employed as the bottom platform for the integration of Si-based monolithic optoelectronic integration circuits (OEICs) [14,15] due to the smaller lattice constant mismatch and similar thermal expansion coefficient with GaAs [13,16], InP [17], and GeSn [18]. High-quality Ge film on large wafer size Si substrate is very important to achieve a Si-based high efficiency light source, large wafer size GaAsOI substrate, InPOI substrate, GeSnOI substrate, and so on.

However, it is very difficult to grow high-quality Ge film on large wafer size Si substrate due to a 4% lattice mismatch and nearly 50% thermal expansion mismatch between Ge and Si, which results in two major problems: (i) high surface roughness with the root mean square (RMS) value above 1 nm due to the Stransky–Krastanov (S–K) growth mode, which hinders the integration with the subsequent active layers [19]; and (ii) high threading dislocation density (TDD) as high as 10^9^ cm^−2^, which will deteriorate device performance [6,12,20]. Therefore, a comprehensive growth optimization study for large wafer scaled and high-quality Ge buffer layer is highly desired for future Si photonics and complementary metal oxide semiconductor (CMOS) integration. In order to achieve high performance Ge-based optoelectronic and electronic devices, a Ge layer on large wafer size Si substrate with flat surface and low defect density is desirable.

Up to now, various reports have exploited growing high-quality Ge films on Si substrate, but they are mostly focused on optimizing a single growth parameter and the influence of all involved factors has not been reported yet. Heteroepitaxy growth is not a new subject but it is a complex one since it involves issues such as strain, defect density, surface roughness, and interfacial quality. Ge is widely used as buffer layer for quantum well structures or as an active material in photonic and electronic devices. Although there is an understanding for the crystal growth mechanism, there is no in-depth analysis or complete research including all the necessary growth optimization and post annealing treatments that have been conducted so far.

In many research reports, a thick graded SiGe buffer layer is grown to control lattice constant mismatch with Si [21,22,23,24]. By using this method, lattice mismatch between Si and Ge changes gradually and a low TDD value with good RMS of 1.02 nm can be obtained [25]. However, this method usually requires the buffer layer with a thickness of 10 μm and Ge composition ranging from 0 to 100%, which are not favorable for the coupling of the optical waveguide with passive devices and high cost for the CMOS integration.

Another approach to grow Ge on Si deals with the control of defect density through a two-step epitaxy. This method involves a low temperature (LT) growth in range of 300–400 °C during which Ge is highly defected and later growth temperature is raised to grow high quality Ge layer. During the first step, the Ge growth follows Stranski–Krastanov mode where a few monolayers of Ge are initially grown, and later nucleation of Ge occurs. The role of LT growth is to promote layer-by-layer growth and to relax the elastic energy in limiting and confining the dislocations without any 3D islanding [26,27]. Then, a high temperature (>500 °C) is employed for the growth of the topmost Ge layer, which offers lower TDD [28]. In addition, the LT Ge layer contains many point defects then the strain relaxation is enhanced via misfit dislocations and LT Ge growth helps to obtain a flat surface. By using such method, although thin (<100 nm) buffer layer is used, a TDD of 9.5 × 10^8^ cm^−2^ is obtained while maintaining a flat surface [29]. In order to further reduce both the TDD and the surface roughness of Ge layers, a post annealing or cycling annealing process was introduced after the LT-Ge or HT-Ge deposition [30,31,32,33] This is due to the thermally assisted glide of the threading arms of misfit dislocations and mutual annihilation or elimination at wafer edges. By using these annealing methods, TDDs can be reduced to 5 × 10^6^ cm^−2^ for the 5 μm thick Ge layer and a relatively lower surface roughness (RMS values are ranging from 0.8 to 2.0 nm) was obtained [34]. Although the TDD and RMS values of these samples are excellent, long annealing time and thick Ge layer are not favorable for industrial applications.

Another approach is to deposit Ge directly on Si substrate and then introduce the annealing step after the Ge growth to reduce the TDD. However, this approach results in a much higher TDD of >10^7^ cm^−2^ [32] and severe inter-mixing occurs at the Si/Ge interface. From the device application perspective, values of TDDs and RMS should be lowered by optimizing the LT-HT two-step Ge growth condition. Further improvement for Ge growth on large wafer size Si substrate might be possible, but it seems rather difficult to achieve both the values of TDDs and RMS below 10^6^ cm^−2^ and 1 nm, respectively.

In this work, a growth analysis of Ge on Si substrate from initial monolayers until the formation of a full Ge buffer up to 2 µm was performed. To obtain the optimized growth conditions for Ge growth, we systematically investigated the influence of LT-Ge buffer layer thickness, HT-Ge growth temperature, total Ge layer thickness, and high temperature thermal treatment on the morphological and crystalline quality of the Ge epilayers. The novelty of this work originates from the strategies used to study the Ge growth and providing a deep understanding in this field for the research community. The outcome of this work gives information about the Ge growth optimization process for future Ge-based photonic and devices on large size Si substrates.

## 2. Materials and Methods

**Figure 1:**
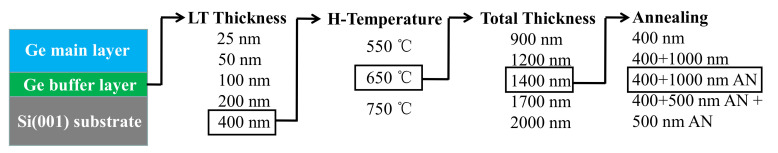
Simplified schematic representation of Ge epilayers on Si substrate.

All Ge epilayers were grown on p-type Si (001) 200 mm wafers in a reduced pressure chemical vapor deposition (RPCVD) ASM Epsilon 2000 reactor (ASM Inc., Almere, The Netherlands). The cleaning methods of Si substrates prior to epitaxy were published before in ref [8]. Based on this conventional growth method [35,36], different growth times, 20 s, 23 s, 30 s, 60 s, 120 s, and 240 s, were applied at 400 °C. Then, at the same growth temperature, we continued to grow different layer thicknesses of Ge ranging from 25 nm, 50 nm, 100 nm, 200 nm, to 400 nm. After the LT-Ge growth, the growth temperature increased up to the target temperature (550 °C, 650 °C, and 750 °C) for HT-Ge growth. The Ge growth was followed from initial wet layer to formation of the nuclide towards 3D growth and 2D growth of Ge. Based on this epi platform HT-Ge layers with different thickness were grown. To further improve the crystal quality for the Ge layer, we performed several annealing processes. The first annealing process was a standard high temperature H_2_ annealing at 820 °C for 10 min compared to the one without annealing. The second annealing process was carried out in two steps: (1) after the first 500 nm HT-Ge growth, high temperature H_2_ annealing was introduced (820 °C for 10 min); (2) step two: if the Ge growth was finished, another high temperature H_2_ annealing was introduced (820 °C for 10 min). Figure 1 represents the simplified schematic of the grown Ge samples.

The crystallographic properties of the Ge layers were examined using high resolution X-ray diffraction (HRXRD), scanning electron microscopy (SEM), atomic force microscopy (AFM), and transmission electron microscopy (TEM) measurements, and optical properties using photoluminescence (PL) characterization. A Bruker JV Delta X X-ray diffractometer was used for crystal and strain analysis of the Ge layer. Atomic force microscope (AFM) Bruker DIMENSION ICON, Inc., Berlin, Germany was performed for Ge surface roughness analysis. Transmission electron microscopy (TEM) Thermo Fisher Talos, Brno, Czech Republic was used to analyze crystalline structure and defects in the Ge layer and dislocations along the Ge/Si interface. The crystal quality of Ge layer was further verified by PL spectra LabRAM HR800, HORIBA jobin Yvon, Paris, France. The photoluminescence (PL) characteristics of the samples were recorded using a 785 nm CW pumping laser and a liquid nitrogen cooled InGaAs detector.

## 3. Results and Discussion

Ge is a strong candidate as a channel material in CMOS, as well as active material in photonic devices for detection or emission of light. It is well known that the performance of these devices is directly related to the material quality. Therefore, this study focused on material improvement and was divided into the following five parts. It begins with analysis of the initial Ge growth when nucleation occurs at LT and then continues to optimize the conditions in both LT and HT epitaxy. This is followed by post annealing treatment at different steps.

### 3.1. Initial Steps of Ge on Si

**Figure 2:**
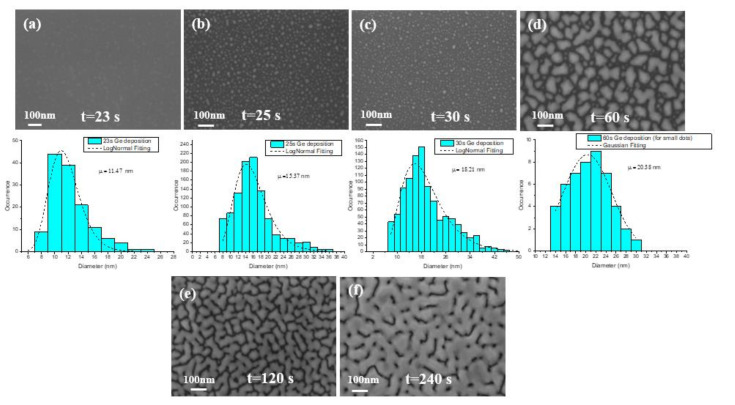
SEM images and histogram for deposition time of Ge (**a**) 23 s, (**b**) 25 s, (**c**) 30 s, (**d**) 60 s, (**e**) 120 s, and (**f**) 240 s.

Since there is a lattice mismatch of 4.2% between Si and Ge, the growth of Ge on Si is governed by Stranski–Krastanov mode. A two-step epi (low and high growth temperature) is demanded for the growth of Ge on Si. The low temperature epi has a vital role for the defect density in the high temperature grown Ge layer. Therefore, in the experiments in this section, the low-temperature epi layer was investigated. The growth temperature was 400 °C whereas the Ge flux was 80 sccm with H_2_ flow of 20 slm. Our observations show that during the initial 20 s of the reaction, a few monolayers of Ge were formed and later the growth transferred into island formation. The evolution of size and distribution of the nucleated sites was investigated in SEM micrographs and histograms in Figure 2a–d. The figure shows that the number of islands constantly increased and with larger size distribution as the deposition continued. Finally, the formed islands began to coalesce and they became larger in size.

**Figure 3:**
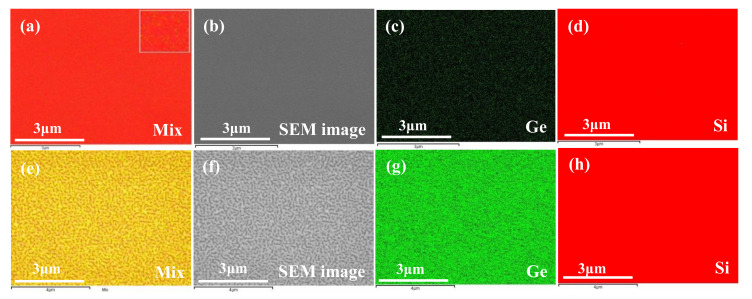
EDS analysis for Ge deposition time of 25 s (**a**–**d**) and 240 s (**e**–**h**). For 25 s, (**a**) mix, (**b**) SEM image, (**c**) extracted result of Ge as epilayer, and (**d**) extracted result of Si as substrate; for 240 s, (**e**) mix, (**f**) SEM image, (**g**) extracted result of Ge as epilayer, and (**h**) extracted result of Si as substrate.

In order to investigate the material distribution, EDS analysis was performed for the layers deposited for 25 s and 240 s as shown in Figure 3a–h. In this analysis, each material has a signature color. Although the chosen color for Ge is black in the first sample to provide highest contrast to the red color Si, in the mixed color micrograph, there is a mostly red color dominant, showing the Si–like surface. For the second sample, the mixed color micrograph is quite different and it has yellow signature which is a mixture of red and green.

**Figure 4:**
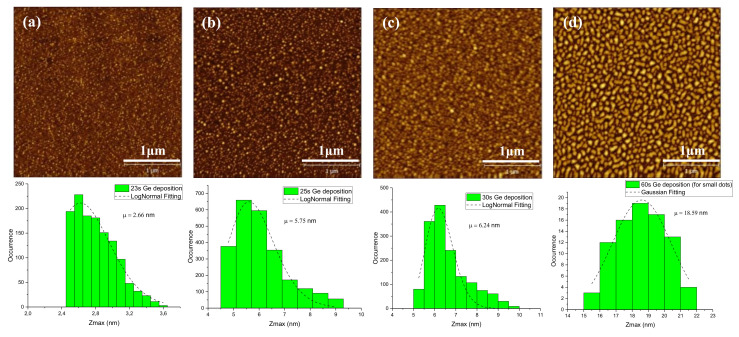
Atomic force microscopy (AFM) images and histogram of Ge height size distributions graphs for deposition time: (**a**) 23 s, (**b**) 25 s, (**c**) 30 s, (**d**) 60 s.

At this stage, AFM analysis was performed on the first four Ge layers in order to obtain more accurate information about the size and shape of islands as shown in Figure 4a–d. Since the surface roughness constantly increases during initial moments of epitaxy, the estimation of this value has no sense. A more accurate observation surface of Ge dots was faceted, and their average size grew 2.66, 5.75, 6.24, to 15.59 nm during the growth 23, 25, 39, to 60 s.

**Figure 5:**
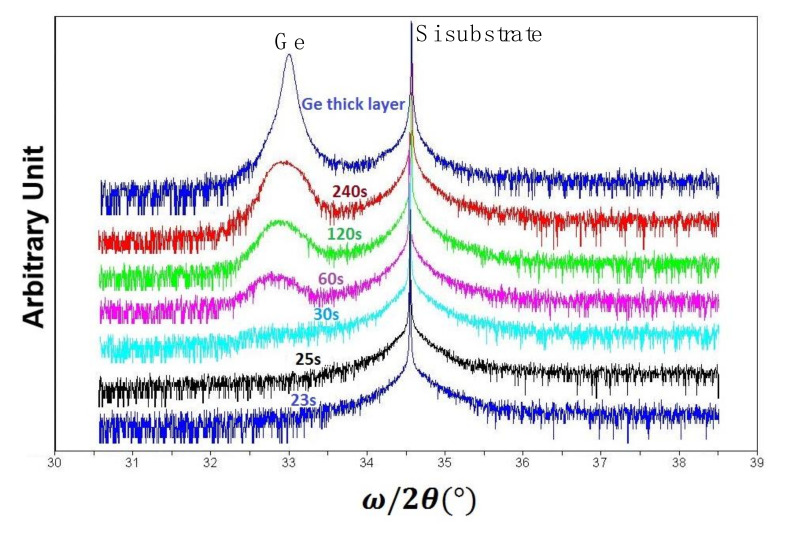
High resolution X-ray diffraction (HRXRD) omega-2theta (004) rocking curves for different Ge deposition time.

Since the small Ge islands are compressively strained, any size increase of Ge islands during the growth may affect their strain. For this reason, the HRXRD omega-2theta rocking curve analysis was performed at (004) reflection as shown in Figure 5. The Ge peak appeared after 30 s deposition time and its intensity constantly increased. Although the acquisition time for the measuring X-ray was 30 s per step, the Ge peak could not be detected during the first 30 s due to low intensity. The full–width half maximum (FWHM) of Ge peak is a signature of the defect density, but in these series of rocking curves, the layer thickness is small and normally the peak broadening is high, making defect density difficult to discuss. However, the relative position of Ge peak to Si substrate peak indicates the strain. The Ge peak had a shift towards the substrate peak from its appearance after 60 s to 240 s. This means that the Ge dots were strained in the beginning and later gradually relaxed with increase of size where after 240 s, the Ge dots were coalesced and a full Ge layer was formed. As we will discuss later, this initial Ge layer was highly defected and Ge layer with higher growth temperatures must be grown in order to improve the layer quality of the Ge buffer layer.

### 3.2. Optimization of LT-Ge Buffer Layer Thickness

**Figure 6:**
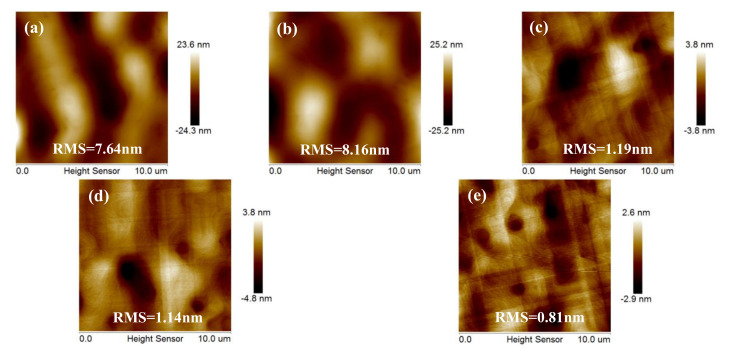
10 × 10 μm^2^ AFM images of 1000 nm high temperature (HT) Ge layers grown on (**a**) 25, (**b**) 50, (**c**) 100, (**d**) 200, and (**e**) 400 nm-thick of low temperature (LT) Ge buffer layers. The RMS of samples were 7.64, 8.16, 1.19, 1.14, and 0.81 nm, respectively.

In order to start the high-quality Ge layers with a rather flat and fully relaxed Ge “seed” layer, we first focused on the thickness optimization for LT-Ge buffer layer. Lower Ge buffer thickness (<40 nm) can easily lead to intermixing between Si and Ge; pure Ge layers with flat surface morphologies were obtained by using thick (>50 nm) LT Ge buffer layers [37]. Therefore, we investigated the growth of LT-Ge buffer layers with different thicknesses (25, 50, 100, 200, and 400 nm) at 400 °C. Then, 1 μm HT-Ge layers were deposited at the temperature of 650 °C. After the HT-Ge growth, typical high temperature H_2_ annealing was carried out at 820 °C for 10 min. Figure 6a–e presents 10 × 10 μm^2^ AFM images from the different LT-Ge layers. For the 25– and 50–nm-thick LT-Ge buffer layers, a high RMS value of 7.64 and 8.16 nm was measured, respectively (in Figure 6a,b). For the LT-Ge buffer below 50 nm, numerous deep holes were observed on the sample’s surface. Meanwhile, the hole’s density decreased, whereas its depth increased with increasing thickness of the LT-Ge buffer layers. That is mainly due to the initial Ge nucleation layer; island formation is not uniform and surface holes will be formed. In comparison, when using 100–, 200– and 400 nm-thick LT Ge buffer layers, the surfaces of the two-step Ge layers were flat with the RMS roughness of 1.19, 1.14, and 0.81 nm, as shown in Figure 6c–e. This indicates the importance of filling of the holes and this may occur when the thickness of the LT–buffer layer is thicker than 100 nm.

**Figure 7:**
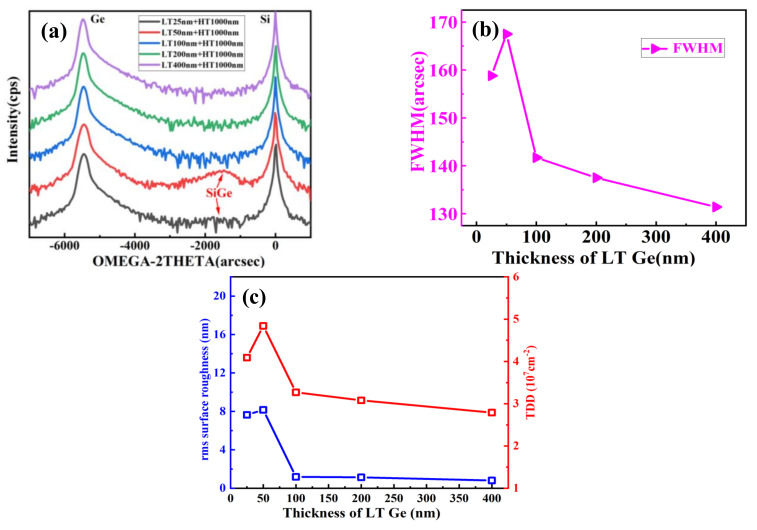
(**a**) HRXRD (004) rocking curves of two-step grown Ge layers using different LT Ge layers, (**b**) the plot of FWHM value changed depending on the thickness of LT Ge buffer layer, and (**c**) RMS and calculated TDD values.

Figure 7a shows the HRXRD rocking curve of (004) Bragg peaks of Ge layer for the increased thickness of the Ge LT-buffer layer from 25 nm to 400 nm. For the two-step grown Ge layers using thin LT Ge buffer layers (25 nm and 50 nm), the Ge peaks were broadened with asymmetric shape where the full width at half maximum (FWHM) values were high (for 25-nm-thick, 159 arc-sec and for 50-nm-thick, 168 arc-sec) in Figure 7b indicating poor Ge quality. In addition, diffused SiGe peaks appeared when the LT layer was below 50-nm-thick, indicating high intermixing of Si into the LT Ge. However, such SiGe peak disappeared with the LT Ge buffer layer thicker than 100 nm thick. We emphasize that all the samples underwent an annealing treatment and the intermixing of Si into Ge was expected. Thus, it was expected that intermixing of Si into Ge would become stronger in the presence of holes for the thin LT buffer layers. When the thickness of LT increased to 100 nm, the FWHM value of Ge decreased sharply, and the narrowest FWHM was 131 arc-sec obtained at 400 nm LT buffer layer, indicating that the highest quality of Ge was obtained at this process. The results are consistent with the AFM image morphology. Considering both TDD and RMS roughness of samples grown at different thicknesses of LT Ge buffer layers as shown in Figure 7c, it suggests that 100 nm Ge LT-buffer layer is the critical value for Ge growth. This explains why when the thick layer is less than 100 nm, the 3D island nucleus in the initial stage of nucleation grows faster than the two-dimensional growth. However, when the thickness of LT Ge is more than 100 nm, the three-dimensional island nucleus tends to become the two-dimensional one, then the flat film gradually forms.

**Figure 8:**
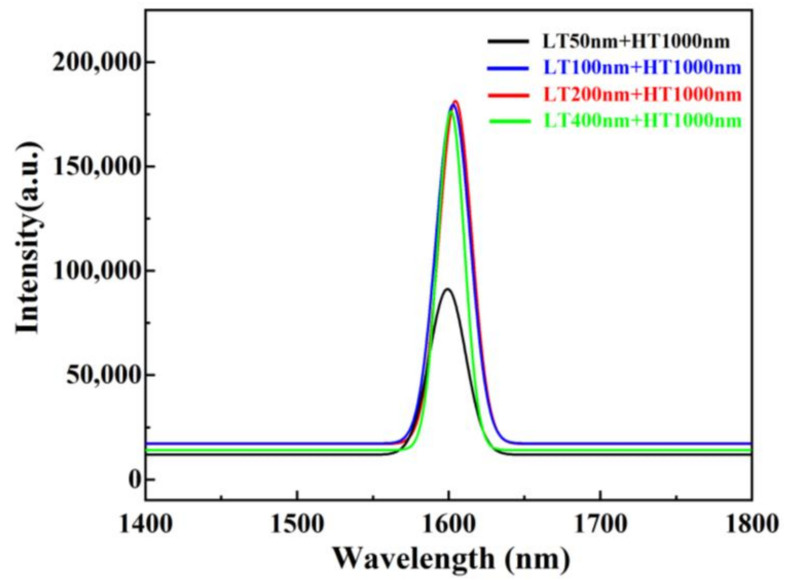
Room temperature of photoluminescence spectrum of two-step grown Ge layers using different thicknesses of LT Ge buffer.

The optical quality of the Ge films with different LT thicknesses were investigated through room temperature PL as shown in Figure 8. The PL spectra of a single-crystalline Ge bulk wafer are shown for comparison. First, the films with LT-Ge thickness above 100 nm exhibited stronger photoluminescence than those of 50 nm, which is indicative of high crystalline quality. This was expected since the TDD and roughness of Ge surface were high when the thickness of LT-Ge layer was less than 50 nm. Second, direct band-to-band luminescence for different samples was observed at 1600 nm (0.775 eV), 1602 nm (0.765 eV), 1604 nm (0.756 eV), and 1598 nm (0.776 eV) responding to 50, 100, 200, and 400 nm LT-Ge thickness. The red-shift of the direct band-to-band luminescence from the expected value of ~0.8 eV may have been caused by the tensile strain of the Ge layer with the different LT-Ge thicknesses, which is related to the change in the Ge band structure. In the growth process, two factors might have affected the Ge band structure and further led to the change in PL spectra. First, tensile strain may have existed due to the thermal mismatch between Ge and Si, and which results in high TDD in Ge layer. Second, Si-Ge intermixing might have occurred when the thickness of LT-Ge was less than 50 nm, which could have likewise resulted in the variation of the band structure of the grown Ge films. Therefore, we can identify that the best quality sample is when the LT-Ge thickness is 400 nm.

### 3.3. Optimization of High Growth Temperature for Ge

**Figure 9:**
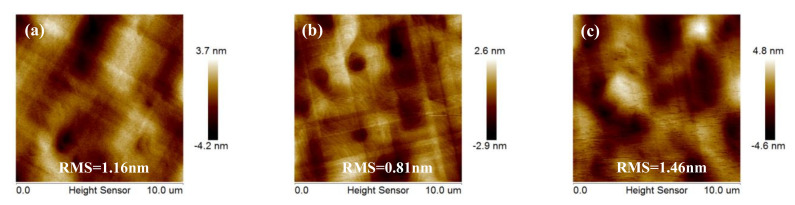
AFM images 10 × 10 μm^2^ of Ge grown at different temperatures: (**a**) 550 °C, (**b**) 650 °C, (**c**) 750 °C. The RMS roughness of the samples were 1.16, 0.81, and 1.46 nm, respectively.

In the hetero-epitaxy process, surface morphology and crystal integrity of materials are affected by the growth temperature (and strain). In general, high growth temperature of Ge is considered in the range of 500–800 °C, when the growth rate is easy to control and the epitaxial layer is uniform. Therefore, the choice of high growth temperatures was 550 °C, 650 °C, and 750 °C in these experiments. Figure 9a–c shows 10 × 10 μm^2^ AFM images of Ge epilayers grown on 400 nm LT–buffer layer at the three above-mentioned growth temperatures.

Analysis of these AFM images indicates a rather rough surface with a little high RMS roughness of 1.16 nm for Ge layer at 550 °C in Figure 9a. A huge number of holes on the surface can be observed. With the increase of the temperature to 650 °C, the surface became smooth and many holes disappeared, the big holes becoming smaller. However, higher growth temperatures can also roughen the surface of the Ge layer, from an RMS roughness of 0.81 nm (650 °C) to 1.26 nm (750 °C) as shown in Figure 9b,c. Figure 9c shows hills (shining point) on the surface.

**Figure 10:**
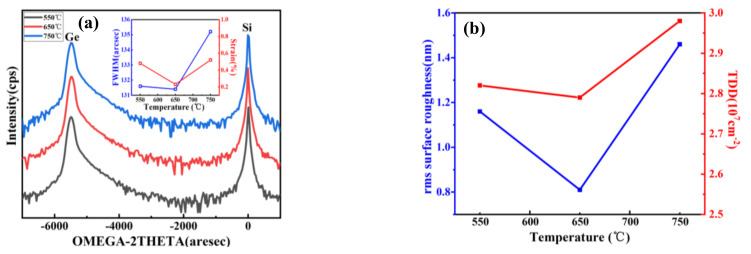
(**a**) HRXRD (004) rocking curves of Ge grow at different temperatures from 550 to 750 °C. The inset shows the calculated strain (right) and FWHM (left) of each Ge curve. (**b**) RMS and calculated TDD values.

Further characterization was focused on the temperature range from 550 to 750 °C. The HRXRD rocking curve of (004) Bragg peaks from two-step grown Ge layers with this temperature range are plotted in Figure 10a. For all samples, the peaks from the Ge buffer appeared at the same angular position, but a slightly right-shifted Ge peak position was clearly observed from −5487 arcsec (650 °C) to −5476 arcsec (550 °C), −5468 arcsec (750 °C). This indicates that a different strain was created in different Ge growth temperatures. Since the strain state of the final Ge epilayer has important consequences for its electrical and optical properties, a HRXRD study was performed to estimate the strain in the epilayer. The generation of the strain was thermally induced in the Ge epilayer during cooling after high temperature growth or annealing temperature to room temperature as Ge and Si have different linear coefficients of thermal expansion (CTE) [38].

The strain in Ge layer can be divided into two parts: (1) compressive strain due to the lattice mismatch between Si and Ge, and (2) tensile strain induced by the cooling process because of the thermal expansion coefficient mismatch. For the different HT Ge layers, the temperatures for the cooling process were different, so the tensile strain induced in these Ge layers were different. We can calculate the perpendicular lattice constant (a^⊥^) using Bragg’s Law as follows: from the peak position of Ge using the Bragg’s law in the form:

(1) $$a^{⊥}=\frac{2\lambda }{sin\left(\frac{\omega }{2}\right)}$$

where *λ* is the wavelength of the incident radiation (Cu Κα1 line, *λ* = 1.5406 Å), and *ω*–*Ge* is the angular position of the Ge peak from the standard (004) *ω*-2θ scan.

(2) $$a^{\left|\right|}=\left(\frac{1+v}{v}\right)\left[a_{Ge}-a⊥\left(\frac{1-v}{1+v}\right)\right]$$

The in-plane lattice constant (ɑ^||^) of the Ge epilayer can be calculated by considering the elastic modulus of Ge, *ν* = 0.271, and unstrained Ge lattice constant, $a_{Ge}$ = 5.65785 Å.

(3) $$Strain\;R\;\%=\frac{a^{\|}-a_{Ge}}{a_{Ge}}$$

The residual tensile strain of the Ge epilayer can hence be estimated using Equation (3).

From the Ge peak positions, the in–plane strain values were 0.48%, 0.23%, and 0.52%, corresponding to the high growth temperature 550 °C, 650 °C, and 750 °C. All the Ge layers were slightly tensile–strained, as in Figure 10a; *R* values decreased with the increase of growth temperature then increased, in the range of 102.3–105.2% 

We also determined the threading dislocation density (TDD) by HRXRD. In HRXRD rocking curve, the FWHM indicates the crystal quality and the broadened diffraction peak implies the increase in the surface and/or interface roughness and bulk-defects. Using Ayers’ theory [39,40], the threading dislocation density D can be calculated from these FWHM values β in arc seconds, as follows:

*D* = 1632 × *β*^2^ (4)

From Figure 10a, the FWHM values are 131.8, 131.2, and 135.2 arcsec; using Equation (4), the TDD values of the Ge layer were calculated to be 2.84 × 10^7^ cm^−2^, 2.78 × 10^7^ cm^−2^, and 2.98 × 10^7^ cm^−2^, corresponding to the grow temperature 550 °C, 650 °C, and 750 °C. Figure 6b shows the plot of RMS roughness from Figure 4 and the TDD values calculated using Equation (4). Similar trends were observed for TDD. The TDD is the main factor to the surface roughness, as the lowest TDD value of 2.78 × 10^7^ cm^−2^ was obtained with an RMS roughness of 0.81 nm at 650 °C HT-Ge growth. Considering both TDD and RMS roughness of samples grown at different temperatures, 650 °C appears to be the optimal growth temperature to lower the TDD whilst retaining a smooth surface.

### 3.4. Optimization of Thickness for the Quality of Ge

**Figure 11:**
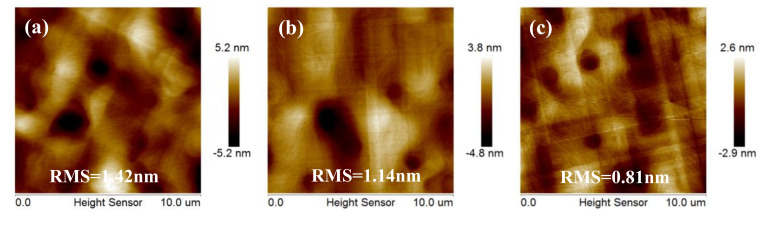

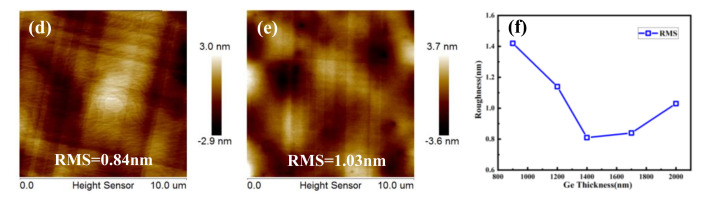
AFM images 10 × 10 μm^2^ of Ge layers grown at 650 °C with thicknesses of (**a**) 900, (**b**) 1200, (**c**) 1400 nm, (**d**)1700 nm, and (**e**) 2000 nm. The RMS roughness of the samples were 1.42, 1.14, 0.81, 0.84, and 1.03 nm, respectively. (**f**) The plot of the RMS roughness of the five samples.

Five different Ge layers were grown with thicknesses 900, 1200, 1400, 1700, and 2000 nm on 400 nm LT-Ge buffer layers with an optimized growth temperature of 650 °C and 820 °C one-step annealing. Figure 11a–e represents 10 × 10 μm^2^ AFM images of Ge layers grown at 650 °C with the above five various thicknesses of Ge. It can be observed that the Ge surface roughness gradually decreased with the increase of film thickness. When the thickness was 1400 nm, the surface roughness reached the minimum of 0.81 nm, and surface roughness could be reduced by half when the thickness was increased by 500 nm. However, as the film thickness continued to increase, the surface roughness gradually increased. Among them, the film thickness increased by 300 nm and the surface roughness increased only by 0.3 nm; when the thickness increased to 2 μm, the surface roughness increased by 24% and was 1.03 nm. Figure 11f shows the plot of the RMS roughness of the five samples. In Figure 11, it can be deduced that the 1400 nm Ge layer had the best quality.

Based on the kinetics of strain relaxation [41], the explanation for these results is that dislocations barely move in the LT layer but can glide easily in the HT layer, dislocations glide and annihilate with the increases of layer thickness, which explains the reduction in RMS roughness for thicker layers. Meanwhile, the strain caused by hetero-growth is completely released with a certain thickness, and surface roughness reaches the lowest value. However, using thicker Ge buffers results in smoother films with the side effect of increasing the bow and stress of the wafer. As the thickness of Ge layer was greater than 1400 nm, a more cross-hatch pattern was likely be encountered which led to an increase of Ge surface roughness and TDD. The most optimal TDD/thickness trade-off was achieved for the thinner layers and the small reduction in TDD of the thicker layers did not justify adding the extra thickness.

### 3.5. Optimization of Post Annealing Conditions

**Figure 12:**
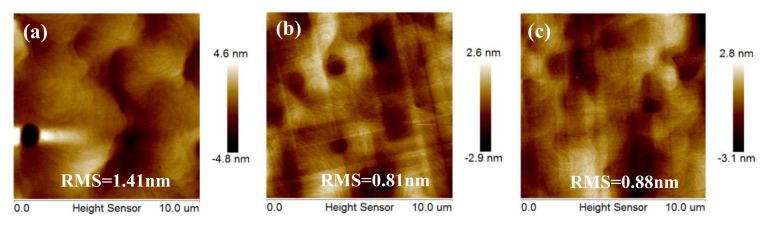
AFM images 10 × 10 μm^2^ of 1.4 μm thick Ge samples (**a**) without annealing (**b**) one-step annealing, and (**c**) two step annealing. RMS values of the Ge surface are (**a**) 1.41 nm, (**b**) 0.81 nm, and (**c**) 0.88 nm, respectively.

The annealing process can significantly relax stress on the epitaxial layer. Based on the above optimized experiment results, the influence of different annealing processes on the surface morphology and strain of Ge film was investigated. In this part, 1.4-μm-thick Ge with 400-nm-thick LT-buffer layer samples grown at 650 °C with the following process (a) without annealing, (b) with one-step annealing, and (c) with two-step annealing were demonstrated. Figure 12a, b shows the surface roughness by 10 × 10 μm^2^ AFM images. Before annealing is introduced, many dislocations and small hills were observed on the surface and the RMS roughness of the Ge surface obtained by AFM was ~1.41 nm as shown in Figure 12a. After one-step annealing, the AFM image in Figure 12b shows a mirror-like surface with RMS roughness of ~0.81 nm. This indicates a significant reduction of ~42.8% in RMS roughness when annealing was introduced after the Ge epitaxial growth. During annealing in the H_2_ environment, surface mobility of the Ge atoms was enhanced and a redistribution of surface atoms was promoted which resulted in a reduction in the final RMS surface roughness.

**Figure 13:**
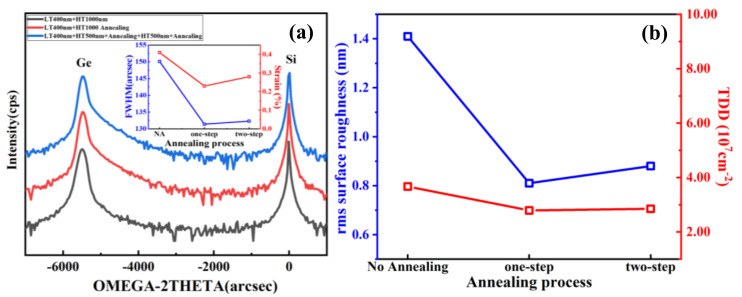
(**a**) HRXRD (004) rocking curves of (LT + HT) Ge grown on Si substrate with different annealing processes: no annealing, one-step annealing, two-step annealing, the inset shows the FWHM left and calculated strain of each sample. The RMS values and the TDD calculated by HRXRD are displayed in (**b**).

Figure 13a shows the HRXRD rocking curve of Ge (004) Bragg peaks from different annealing processes. We found the Ge positions were −5477 arcsec, −5481 arcsec and −5472 arcsec for the three processes of no annealing, one-step annealing, and two-step annealing. Using the above Equation, we can calculate the tensile-strained R values, which were 0.31%, 0.23%, and 0.29%, corresponding to the three annealing conditions, respectively, and the trend of strain can be seen in Figure 13a. This is due to differences in thermal expansion coefficients between Ge and Si. Meanwhile, the change of Ge FWHM values also reflects the change in the quality of Ge via different annealing conditions. Figure 13b shows the plot of RMS roughness and the TDD calculated by HRXRD against the variation of the annealing process. It can be seen that the RMS roughness of the Ge film had a sharp drop after one-step annealing, with the highest (~1.41 nm) and lowest (~0.81 nm) RMS roughness. In the subsequent growth, the two-step annealing process was selected since it resulted in a slightly higher RMS roughness as shown in Figure 13b. This was also reflected in the TDD results where the one-step annealing of the Ge film was lowest with a TDD value of 2.79 × 10^7^ cm^−2^.

**Figure 14:**
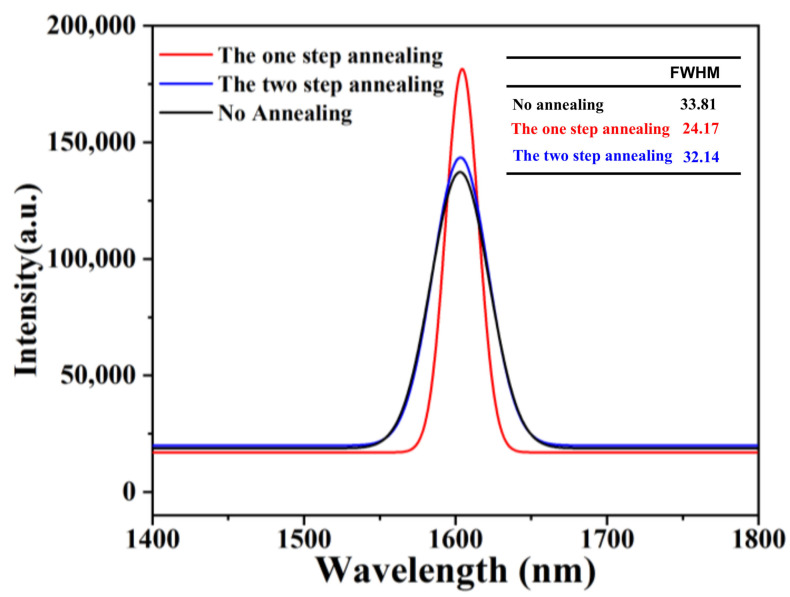
Room temperature of photoluminescence spectrum of two-step grown 1.4 μm Ge layers using different annealing processes.

Figure 14 demonstrates the PL results acquired at room temperature. It is clear from the figure that the sample of the one-step annealing process showed the highest PL intensity and narrowest full-width at half-maximum (FWHM), which indicates good crystal quality of the Ge layer with fewer TDDs and less carrier scattering. Comparing with the no annealing process, the annealing process can obviously improve the quality of the film performance with the intensity peak increasing strongly and FWHM value decreasing significantly (from 33.81 nm to below 24.17 nm). However, the two-step annealing process can deteriorate the optical property of Ge layer performance with intensity peak decreasing. This phenomenon is mainly attributed to the effect of the annealing process on Ge material quality. The one-step annealing process can make the TD glide and annihilate, which leads to a perfect surface morphology. However, the two-step annealing causes significant Ge–Si intermixing at the Ge/Si interface and the TDs are pushed upward into the Ge layer.

**Figure 15:**
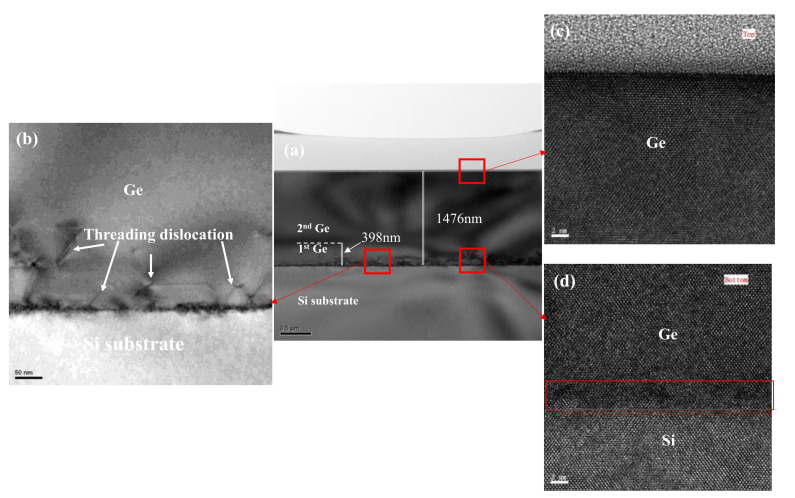
(**a**) Cross-sectional dark field TEM images of an optimized process of 1.4 μm–thick Ge layer with the thickness of a 400 nm LT–buffer layer grown on a Si substrate; (**b**) high resolution TEM images of the first step Ge/Si interface of (**a**). Many threading dislocations appear at the interface of Ge/Si and disappear as the Ge buffer increases. HR-TEM images of two selected defects, the top Ge layer pointed out by an arrow in (**c**) and near the Ge/Si interface highlighted by red frame in (**d**).

Through the above process optimization, we obtained the optimized process parameters of high-quality Ge film as follows: the thickness of low temperature Ge buffer was 400 nm, high growth temperature was 650 °C, the total thickness of Ge layer was 1400 nm, with one-step annealing at 820 °C for 10 min. To confirm the quality of the obtained Ge film manufactured by above optimized process, a TEM study was performed. Figure 15 presents the cross-sectional TEM images of the Ge film. Figure 15a shows the cross-sectional images of the Ge on Si substrate; the thicknesses of the 1st Ge buffer layer and the total Ge layer were measured to be 400 nm and 1400 nm. Figure 15b is the magnification of the dislocation in Figure 15a highlighted by red frame. Many TDs are visible near the interface, the {111} stacking faults (SF) observed in Figure 15b, which are related to presence of accumulated misfit dislocations, but the TDD decreases with the increasing thickness of the Ge layer due to defect “healing”. Most of the threading dislocations lay in the growth of Ge LT-buffer layer and were concentrated in the 100 nm vicinity of the interfaces with Si substrate. Figure 15c,d are the high resolution TEM (HR-TEM) images of the top Ge layer and Ge/Si interface which are highlighted by red frame. In Figure 15c, a single crystal high quality Ge epitaxy with few defects can be seen from the regular lattice periodic arrangement. Figure 15d shows a distortion of the periodic lattice arrangement in the Ge/Si interface, which reveals stacking faults along the (111) planes and misfit dislocations.

**Figure 16:**
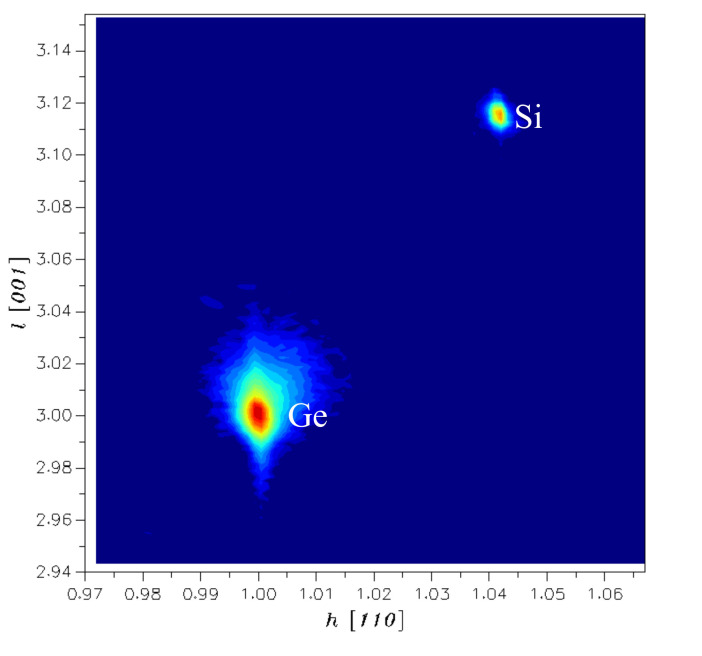
High–resolution reciprocal lattice maps (HRRLMs) around Si and Ge (113) diffraction of the optimized process of 1.4 μm–thick Ge layer.

In order to further discuss the crystallinity of the Ge layer obtained by the above optimized process, high-resolution reciprocal lattice maps (HRRLMs) around (113) reflection were performed. Figure 16 shows the result of a steeper decay in h [110] direction and a stronger tail in l [001] towards the Si peak. In l [001] direction, the position of the Ge peak was slightly shifted, which indicates that the optimized Ge layer exhibited lower TDD. There was a slight tensile strain in the Ge diffusion between the interface due to the different thermal expansion coefficients of Si and Ge. Meanwhile, in order to verify the high quality of Ge, we calculated the TDD of optimized Ge film by etch pit density (EPD) experiment. The obtained TDD value was 3.8 × 10^7^ cm^−2^ by counting the number of etch pits, which is close to the HRXRD outcome.

Then, the final recipe after the critical growth optimization is summarized as: LT-Ge thickness should be around 400 nm, and for HT Ge, 1400 nm Ge layer followed by one-step annealing at 820 °C of 10 min. The properties of Ge materials prepared by this optimized process are as follows: RMS = 0.81 nm, TDD = 2.79 × 10^7^ cm^−2^, tensile strain 0.32%. It is emphasized here that this outcome presents the best values for Ge growth using the above conditions but there is also a tolerance level for both layer thickness and the annealing temperature.

## 4. Conclusions

This work presented the growth of high epitaxial quality Ge layer on Si by applying a two-step epitaxy (LT and HT) by using the RPCVD technique. The study started from the initial nucleation steps of Ge on Si at LT and later, it extended to layer-by-layer growth for a thicker Ge layer. In these experiments, the parameters, e.g., layer thickness, growth temperature of HT Ge growth and post annealing treatment, were optimized to decrease TDD and surface roughness of the Ge layers. A meaningful TDD value for the Ge layer was as low as 2.78 × 10^7^ cm^−2^ with the surface roughness of 0.8 nm when the LT-Ge buffer layer was 400 nm and total thickness was 1400 nm at 650 °C. In order to further improve the material quality, one-step annealing and two-step annealing were carried out at 820 °C in the H_2_ atmosphere. After one-step annealing, TDD was further reduced by 30% compared to that of as-deposited samples which also displayed a low tensile strain of 0.23%. Meanwhile, PL results show that one-step annealing has a stronger peak density than others, which displays a narrow FWHM of 24.17 nm. From our results, optimal process parameters of high quality of Ge layer are the following: high growth temperature is 650 °C, one-step annealing at 820 °C for 10 min, the thickness of low temperature Ge buffer is 400 nm, and the total thickness of Ge layer is 1400 nm. This research provides not only very meaningful conclusions for the epitaxy of Ge on Si, but also provides an important support for the application of high-quality Ge films on large-size silicon substrates in microelectronics and optoelectronics.

## Data Availability

The data presented in this study are available on request from the corresponding authors.

## References

[1] Zhao X., Moeen M., Toprak M.S., Wang G., Luo J., Ke X., Li Z., Liu D., Wang W., Zhao C. (2019). Design impact on the performance of Ge PIN photodetectors. J. Mater. Sci. Mater. Electron..

[2] Feng N.-N., Feng D., Liao S., Wang X., Dong P., Liang H., Kung C.-C., Qian W., Fong J., Shafiiha R. (2011). 30GHz Ge electro-absorption modulator integrated with 3μm silicon-on-insulator waveguide. Opt. Express.

[3] Hartmann J.M., Abbadie A., Papon A.M., Holliger P., Rolland G., Billon T., Fédéli J.M., Rouvière M., Vivien L., Laval S. (2004). Reduced pressure–chemical vapor deposition of Ge thick layers on Si(001) for 1.3–1.55-μm photodetection. J. Appl. Phys..

[4] Radamson H., Thylén L. (2015). Monolithic Nanoscale Photonics–Electronics Integration in Silicon and Other Group IV Elements.

[5] Chaisakul P., Marris-Morini D., Frigerio J., Chrastina D., Rouifed M.-S., Cecchi S., Crozat P., Isella G., Vivien L. (2014). Integrated germanium optical interconnects on silicon substrates. Nat. Photon.

[6] Radamson H.H., Zhang Y., He X., Cui H., Li J., Xiang J., Liu J., Gu S., Wang G. (2017). The Challenges of Advanced CMOS Process from 2D to 3D. Appl. Sci..

[7] Radamson H.H., Zhu H., Wu Z., He X., Lin H., Liu J., Xiang J., Kong Z., Xiong W., Li J. (2020). State of the Art and Future Perspectives in Advanced CMOS Technology. Nanomaterials.

[8] Wang G., Kolahdouz M., Luo J., Qin C., Gu S., Kong Z., Yin X., Xiong W., Zhao X., Liu J. (2019). Growth of SiGe layers in source and drain regions for 10 nm node complementary metal-oxide semiconductor (CMOS). J. Mater. Sci. Mater. Electron..

[9] Saraswat K., Chui C.O., Krishnamohan T., Kim D., Nayfeh A., Pethe A. (2006). High performance germanium MOSFETs. Mater. Sci. Eng. B.

[10] Radamson H.H., Kolahdouz M. (2015). Selective epitaxy growth of Si1−xGex layers for MOSFETs and FinFETs. J. Mater. Sci. Mater. Electron..

[11] CorClaeys C., Simoen E. (2017). Germanium-Based Technologies: From Materials to Devices.

[12] Radamson H.H., Simoen E., Luo J., Zhao C. (2018). Past, Present and Future of CMOS.

[13] Wang X., Xiang J., Han K., Wang S., Luo J., Zhao C., Ye T., Radamson H.H., Simoen E., Wang W. (2017). Physically Based Evaluation of Effect of Buried Oxide on Surface Roughness Scattering Limited Hole Mobility in Ultrathin GeOI MOSFETs. IEEE Trans. Electron Devices.

[14] Abouzaid O., Mehdi H., Martin M., Moeyaert J., Salem B., David S., Souifi A., Chauvin N., Hartmann J.-M., Ilahi B. (2020). O-Band Emitting InAs Quantum Dots Grown by MOCVD On A 300 mm Ge-Buffered Si (001) Substrate. Nanomaterials.

[15] Xiong W., Wang G., Du Y., Lin H., Zhao X., Yu J., Kong Z., Dong Y., Jiang H., Tao Y. (2021). Integration of silicon nitride waveguide in Ge-on-insulator substrates for monolithic solutions in optoelectronics. J. Mater. Sci. Mater. Electron..

[16] Bogumilowicz Y., Hartmann J., Rochat N., Salaun A., Martin M., Bassani F., Baron T., David S., Bao X.-Y., Sanchez E. (2016). Threading dislocations in GaAs epitaxial layers on various thickness Ge buffers on 300 mm Si substrates. J. Cryst. Growth.

[17] Merckling C., Waldron N., Jiang S., Guo W., Richard O., Douhard B., Moussa A., Vanhaeren D., Bender H., Collaert N. (2013). Selective area growth of InP in shallow trench isolation on large scale Si(001) wafer using defect confinement technique. J. Appl. Phys..

[18] Alharthi B., Dou W., Grant P.C., Grant J.M., Morgan T., Mosleh A., Li B., Mortazavi M., Naseem H., Yu S.-Q. (2019). Low temperature epitaxy of high-quality Ge buffer using plasma enhancement via UHV-CVD system for photonic device applications. Appl. Surf. Sci..

[19] Michel J., Liu J., Kimerling L.C. (2010). High-performance Ge-on-Si photodetectors. Nat. Photon..

[20] Halbwax M., Rouviere M., Zheng Y., Débarre D., Nguyen L.H., Cercus J.-L., Clerc C., Yam V., Laval S., Cassan E. (2005). UHV-CVD growth and annealing of thin fully relaxed Ge films on (001)Si. Opt. Mater..

[21] Yoon T.-S., Liu J., Noori A.M., Goorsky M.S., Xie Y.-H. (2005). Surface roughness and dislocation distribution in compositionally graded relaxed SiGe buffer layer with inserted-strained Si layers. Appl. Phys. Lett..

[22] Bogumilowicz Y., Hartmann J., Di Nardo C., Holliger P., Papon A.-M., Rolland G., Billon T. (2006). High-temperature growth of very high germanium content SiGe virtual substrates. J. Cryst. Growth.

[23] Yamamoto Y., Zaumseil P., Schubert M.A., Tillack B. (2018). Influence of annealing conditions on threading dislocation density in Ge deposited on Si by reduced pressure chemical vapor deposition. Semicond. Sci. Technol..

[24] Yamamoto Y., Corley C., Schubert M.A., Zoellner M., Tillack B. (2021). Threading Dislocation Reduction of Ge by Introducing a SiGe/Ge Superlattice. ECS J. Solid State Sci. Technol..

[25] Tao K., Wang J., Jiang S., Jia R., Jin Z., Liu X. (2019). High-quality Ge-rich SiGe thin films epitaxially grown on Si at low temperature by a two-step approach. CrystEngComm.

[26] Nayfeh A., Chui C.O., Saraswat K.C., Yonehara T. (2004). Effects of hydrogen annealing on heteroepitaxial-Ge layers on Si: Surface roughness and electrical quality. Appl. Phys. Lett..

[27] Miao Y.-H., Hu H.-Y., Li X., Song J.-J., Xuan R.-X., Zhang H.-M. (2017). Evaluation of threading dislocation density of strained Ge epitaxial layer by high resolution x-ray diffraction. Chin. Phys. B.

[28] Kim H.-W., Shin K.W., Lee G.-D., Yoon E. (2009). High quality Ge epitaxial layers on Si by ultrahigh vacuum chemical vapor deposition. Thin Solid Films.

[29] Sakai A., Tatsumi T. (1994). Ge growth on Si using atomic hydrogen as a surfactant. Appl. Phys. Lett..

[30] Hartmann J., Damlencourt J.-F., Bogumilowicz Y., Holliger P., Rolland G., Billon T. (2005). Reduced pressure-chemical vapor deposition of intrinsic and doped Ge layers on Si(001) for microelectronics and optoelectronics purposes. J. Cryst. Growth.

[31] Tan Y., Tan C. (2012). Growth and characterization of germanium epitaxial film on silicon (001) using reduced pressure chemical vapor deposition. Thin Solid Films.

[32] Lee K.H., Bao S., Chong G.Y., Tan Y.H., Fitzgerald E.A., Tan C.S. (2015). Defects reduction of Ge epitaxial film in a germanium-on-insulator wafer by annealing in oxygen ambient. APL Mater..

[33] Hartmann J., Abbadie A., Barnes J., Fédéli J., Billon T., Vivien L. (2010). Impact of the H2 anneal on the structural and optical properties of thin and thick Ge layers on Si; Low temperature surface passivation of Ge by Si. J. Cryst. Growth.

[34] Hartmann J., Aubin J. (2018). Assessment of the growth/etch back technique for the production of Ge strain-relaxed buffers on Si. J. Cryst. Growth.

[35] Wang G., Luo J., Qin C., Liang R., Xu Y., Liu J., Li J., Yin H., Yan J., Zhu H. (2017). Integration of Highly Strained SiGe in Source and Drain with HK and MG for 22 nm Bulk PMOS Transistors. Nanoscale Res. Lett..

[36] Wang G., Metzler J.B. (2019). Investigation on SiGe Selective Epitaxy for Source and Drain Engineering in 22 nm CMOS Technology Node and Beyond.

[37] Shin K.W., Kim H.-W., Kim J., Yang C., Lee S., Yoon E. (2010). The effects of low temperature buffer layer on the growth of pure Ge on Si(001). Thin Solid Films.

[38] Ni W.-X., Ekberg J., Joelsson K., Radamson H., Henry A., Shen G.-D., Hansson G. (1995). A silicon molecular beam epitaxy system dedicated to device-oriented material research. J. Cryst. Growth.

[39] Ayers J. (1994). The measurement of threading dislocation densities in semiconductor crystals by X-ray diffraction. J. Cryst. Growth.

[40] Hansson G.V., Radamsson H.H., Ni W.-X. (1995). Strain and relaxation in Si-MBE structures studied by reciprocal space mapping using high resolution X-ray diffraction. J. Mater. Sci. Mater. Electron..

[41] Wang T., Zhang Y.-W., Chua S. (2001). Dislocation evolution in epitaxial multilayers and graded composition buffers. Acta Mater..

